# Editorial: Highlights of 1st International Conference on Sustainable and Intelligent Phytoprotection (ICSIP 2025)

**DOI:** 10.3389/fpls.2026.1823909

**Published:** 2026-04-20

**Authors:** Neil Vaughan, Ye Liu, Chengcheng Chen, Wen-Hao Su, Alvaro Fuentes

**Affiliations:** 1 Department of Clinical and Biomedical Sciences (CBS), University of Exeter, Exeter, United Kingdom; 2 School of Artificial Intelligence, Nanjing Agricultural University, Nanjing, Jiangsu, China; 3 Department of Agricultural Engineering, College of Computer Science, Shenyang Aerospace University, Shenyang, China; 4 China Agricultural University, Beijing, China; 5 Department of Electronics Engineering, Jeonbuk National University , Jeonju, Republic of Korea

**Keywords:** ecological, farming, intelligent, phytoprotection, sustainable

## 1 Introduction

The International Conference on Sustainable and Intelligent Phytoprotection (ICSIP 2025) was conceived at a pivotal moment in the evolution of plant protection science. For decades, phytoprotection has been grounded primarily in chemistry, biology, and ecology. Today, however, the convergence of these disciplines with Information and Communication Technology (ICT) is transforming both the theoretical foundations and practical applications of crop protection. This Research Topic, aligned with the conference theme of “Sustainable and Intelligent Phytoprotection,” captures that transformation and showcases how digital innovation is reshaping agricultural resilience worldwide, including contributions in 14 original articles including 82 authors worldwide. The hyperlink to the Research Topic is *Highlights of 1st International Conference on Sustainable and Intelligent Phytoprotection*, so readers can easily access and navigate to the full Research Topic.

## 2 Framing the vision: from conventional protection to intelligent systems

The ICSIP 2025 emphasized a paradigm shift: the emergence of “Intelligent Phytoprotection” as a dynamic interdisciplinary field. Contributors were invited to explore how technologies such as satellite remote sensing, radar detection, UAV monitoring, aerial image processing, Internet of Things (IoT) networks, big data analytics, blockchain, and artificial intelligence (AI) can be harnessed to achieve sustainable plant protection outcomes.

## 3 Thematic synthesis of contributing articles: toward an integrated intelligent phytoprotection ecosystem

**Figure 1:**
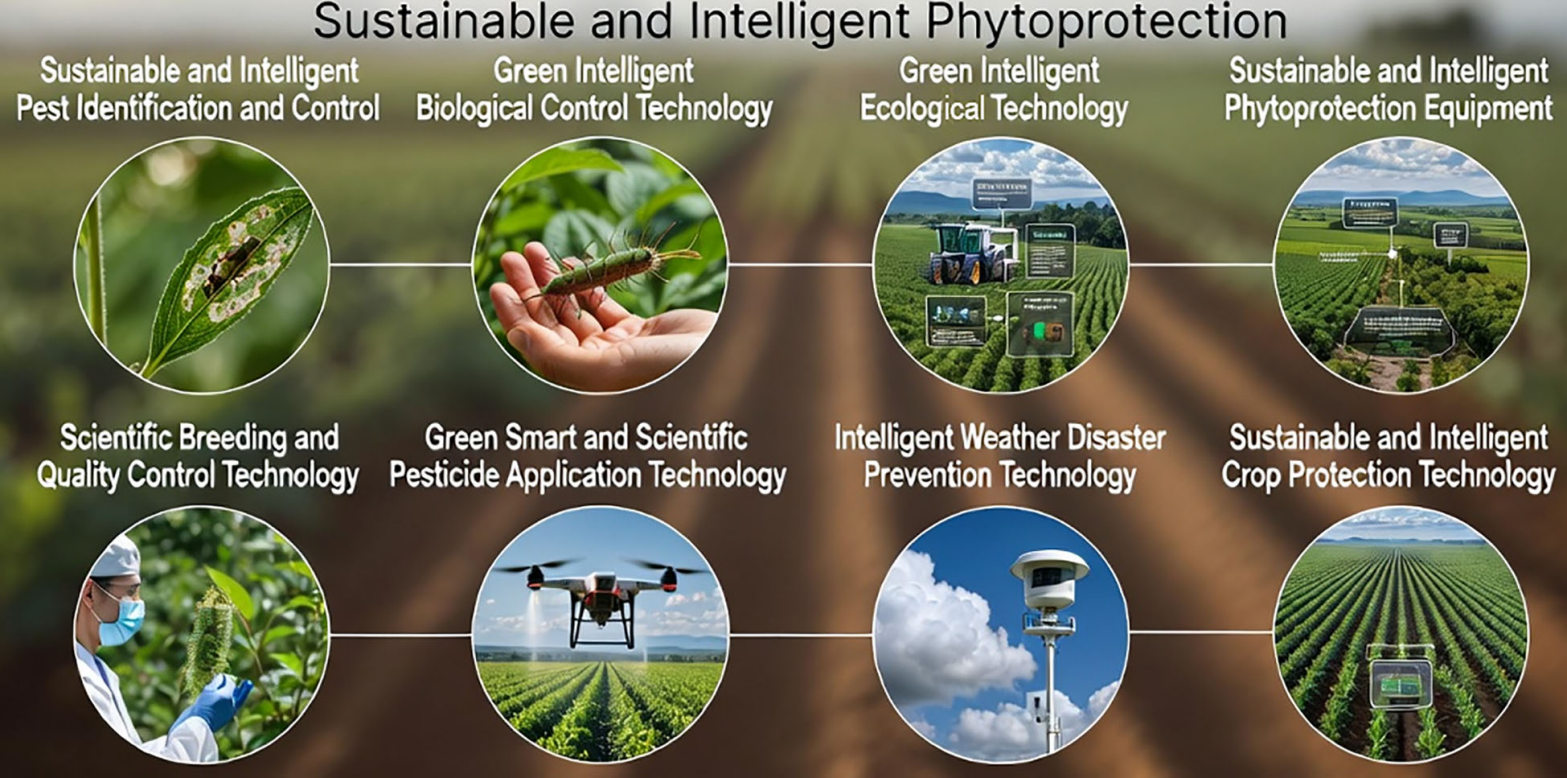
Several novel emerging themes within research articles in this research topic of Sustainable and Intelligent Phytoprotection.

The articles collected in this Research Topic collectively illustrate a decisive transition in phytoprotection, from isolated algorithmic innovations to an interconnected ecosystem of sensing, cognition, decision-making, and autonomous execution. The contributions cluster into four interrelated research domains: (1) intelligent perception and fine-grained recognition, (2) lightweight and data-efficient learning strategies, (3) spatial modeling and structural quantification, and (4) autonomous systems and robotic actuation. Together, they define the computational backbone of sustainable and intelligent phytoprotection. Several of these novel emerging themes are present within research articles in this topic of Sustainable and Intelligent Phytoprotection, shown in **Figure 1**.

### 3.1 Intelligent perception: fine-grained detection and disease recognition

A dominant category across the Research Topic centers on high-precision visual perception for crops, pests, and diseases. This category includes these articles in this ICSIP Research Topic:

- Study on automatic detection of wheat spike grain number based on deep learning (Zang et al.).
- YOLO-LitchiVar: a lightweight and high-precision detection model for fine-grained litchi variety identification (Xu et al.).
- Identification of tobacco leaf diseases using hyperspectral imaging and machine learning with SHAP interpretability analysis (Luo et al.).
- A lightweight intelligent grading method for lychee anthracnose (Xu et al.).
- YOLO-lychee-advanced: an optimized detection model for lychee pest damage (Wu et al.).
- AMS-YOLO: multi-scale feature integration for intelligent plant protection against maize pests (Deng et al.).
- Lightweight grading method for potato late blight severity (Yuan et al.).

These works share a common ambition: translating complex plant phenotypes into computationally measurable features. Several novel articles converge methodologically around multi-scale convolutional architectures (YOLO variants, UNet integrations, attention modules) while diverging in crop species and task specificity. The technical trend is clear: detection is becoming more fine-grained, lightweight, and field-deployable.

Two broader insights emerge: (1) From detection to quantification: Grain counting in wheat and severity grading in disease studies signal a shift from binary classification toward agronomically meaningful metrics. (2) From accuracy to interpretability: The tobacco hyperspectral study (Luo et al.) integrates SHAP analysis, addressing explainability, which is a key limitation in AI-driven phytoprotection. This reflects maturation of the field from “black-box accuracy” to “trustworthy intelligence.” Collectively, these perception-focused works establish the sensory layer of intelligent phytoprotection systems.

### 3.2 Data-efficient and generative intelligence: learning under constraints

A second cluster addresses a fundamental agricultural challenge: limited labeled data and high annotation costs. These works tackle the data bottleneck from complementary angles: (1) Synthetic data augmentation (GAN-based generation) enhances rare disease sample availability. (2) Knowledge distillation and semi-supervised learning improve performance with reduced labeling effort. (3) Curated datasets provide foundational infrastructure for training and benchmarking. This cluster signifies a strategic pivot in intelligent phytoprotection research, from maximizing model complexity to optimizing learning efficiency. In resource-constrained agricultural environments, computational frugality and scalable data strategies are prerequisites for real-world adoption. Key contributions include:

- TeaWeeding-Action: a vision-based dataset for weeding behavior recognition (Han et al., 2025).
- KD-SSGD: knowledge distillation-enhanced semi-supervised germination detection (Chen et al.).
- SinGAN-CBAM: a multi-scale GAN with attention for few-shot plant disease image generation (Wu et al.).

### 3.3 Structural quantification and spatial intelligence

Beyond surface-level recognition, intelligent phytoprotection increasingly relies on structural and spatial modeling of crop environments. These studies represent a move from 2D image-based detection to 3D and spectral-dimensional understanding. LiDAR-derived canopy volume estimation provides actionable metrics for precision spraying, pruning, and yield modeling. This category expands phytoprotection from reactive detection to spatially optimized intervention, integrating digital twins of crop architecture into decision support systems. Notable contributions include:

- Identification of tobacco leaf diseases using hyperspectral imaging and machine learning with SHAP interpretability analysis (Luo et al.).
- Calculation method of canopy effective volume based on LiDAR point cloud data (Ma et al.).

### 3.4 Autonomous systems and robotic intelligence

The most system-level innovations appear in contributions focused on robotics and UAV platforms. These works shift the focus from “seeing” to “acting.” Key thematic alignments include: (1) Robust control under environmental disturbances, ensuring UAV stability in wind conditions (Zhu et al.). (2) Multi-objective optimization, balancing efficiency, obstacle avoidance, and energy use in dynamic fields (Yang et al.). (3) Human–robot interaction modeling, enhancing collaborative robotic harvesting systems (Yao et al.). This cluster completes the intelligent phytoprotection loop: (i) perception (ii) decision (iii) autonomous execution. Articles include:

- Prescribed time backstepping sliding mode control for attitude stabilization of plant-protection UAVs under wind and motor disturbances. (Zhu et al.).
- AgriPath: a robust multi-objective path planning framework for agricultural robots in dynamic field environments (Yang et al.).
- Research on the method of shiitake mushroom picking robot based on CSO-ASTGCN human action prediction network (Yao et al.).

## Intelligent pest identification and precision control

A central theme emerging from the published contributions is the development of sustainable and intelligent pest identification and control systems. Multiple studies illustrate the application of deep learning models for real-time pest recognition using UAV-captured imagery and ground-based sensor networks. These systems markedly improve early detection accuracy while reducing reliance on blanket pesticide applications.

## Green intelligent biological and ecological control technologies

Another major axis of this Research Topic is the advancement of green biological and ecological control technologies. The collected works highlight innovations in microbial agents, beneficial insect deployment, and habitat-based ecological engineering, all enhanced by digital monitoring systems.

## Smart pesticide application and equipment innovation

Precision pesticide application technologies feature prominently among the contributions. Researchers present smart spraying systems equipped with computer vision modules capable of distinguishing crop from weed or diseased foliage in real time. Variable-rate application algorithms significantly reduce chemical usage while maintaining crop protection efficacy.

## Scientific breeding, quality control, and data infrastructure

Beyond direct pest control, several articles expand the scope of intelligent phytoprotection to include scientific breeding and quality control technologies. High-throughput phenotyping systems, combined with machine learning algorithms, accelerate the identification of pest-resistant cultivars.

## Intelligent weather disaster prevention and climate resilience

In the context of intensifying climate variability, intelligent weather disaster prevention technologies have emerged as a critical focus. Articles within this Research Topic employ satellite remote sensing and radar-based monitoring to model extreme weather risks affecting crop health. Predictive analytics frameworks integrate meteorological data with crop growth models to anticipate disease outbreaks linked to humidity, temperature, and storm events.

## Interdisciplinary convergence and collaborative ecosystems

One significant achievement of this Research Topic is its demonstration of effective interdisciplinary integration. Computer scientists, agronomists, ecologists, engineers, and industry practitioners have contributed complementary expertise, reflecting the collaborative ethos emphasized in the ICSIP 2025 call for papers.

## Positioning within the broader research landscape

Taken together, these contributions situate intelligent phytoprotection at the intersection of: (1) Computer vision and deep learning, (2) Agricultural robotics and control engineering, (3) Remote sensing and spatial modelling, (4) Data science and sustainable agronomy. The field is evolving from discipline-specific innovation toward a cyber-physical agricultural ecosystem, where sensors, AI models, robotic agents, and ecological principles operate synergistically. Importantly, the Research Topic reveals a maturing trajectory: Phase 1: Detection accuracy improvements. Phase 2: Lightweight and interpretable AI. Phase 3: Data-efficient and generative frameworks. Phase 4: Full autonomous and precision-integrated systems. The collected works span all four phases, indicating both depth and systemic breadth.

## Broader implications and future directions

The findings presented in this Research Topic resonate far beyond the academic sphere. Sustainable and intelligent phytoprotection directly contributes to global food security, environmental conservation, and rural economic stability. By reducing chemical inputs, improving resource efficiency, and enhancing resilience to climate and biological threats, intelligent systems can help align agricultural productivity with the Sustainable Development Goals. Future research challenges warrant continued attention: (1) Data interoperability and standardization, to ensure seamless integration across platforms and regions. (2) Scalability and affordability, particularly for smallholder farming systems. (3) Ethical governance and data security, especially in the use of blockchain and AI-driven decision systems. (4) Capacity building and knowledge transfer, bridging technological advances with farmer adoption.The articles within this Research Topic provide both technical solutions and strategic insights that address these challenges, laying the groundwork for future research and policy development.

## Conclusion

This Research Topic affirms that sustainable and intelligent phytoprotection is not a distant aspiration but an actively unfolding reality. By integrating ecological wisdom with digital intelligence, the contributions presented here illuminate a path toward more efficient, environmentally conscious, and resilient agricultural systems. ICSIP 2025 has served as a vital catalyst for this transformation, fostering dialogue across disciplinary and geographical boundaries. The work assembled in this Research Topic exemplifies the innovative spirit and collaborative determination necessary to secure the vitality and longevity of global agricultural ecosystems. As intelligent phytoprotection continues to evolve, the foundations laid by ICSIP will support the next generation of research, technology, and practice in sustainable crop protection.
